# Ethical Diversity and Practical Uncertainty: A Qualitative Interview Study of Clinicians’ Experiences in the Implementation Period Prior to Voluntary Assisted Dying Becoming Available in their Hospital in Victoria, Australia

**DOI:** 10.1007/s11673-022-10224-5

**Published:** 2023-02-17

**Authors:** Rosalind McDougall, Bridget Pratt, Marcus Sellars

**Affiliations:** 1 Centre for Health Equity, Melbourne School of Population and Global Health, University of Melbourne, Melbourne, Victoria 3010 Australia; 2 Queensland Bioethics Centre, Australian Catholic University, Brisbane, Queensland 4000 Australia; 3 Department of Health Services Research & Policy, Research School of Population Health, College of Health & Medicine, The Australian National University, Australia, Canberra, Australian Capital Territory 2600 Australia

**Keywords:** Voluntary assisted dying, Euthanasia, Implementation, Diversity

## Abstract

In the Australian state of Victoria, legislation allowing voluntary assisted dying (VAD) passed through parliament in November 2017. There was then an eighteen-month period before the start date for patient access to VAD, referred to as the “implementation period.” The implementation period was intended to allow time for the relevant government department and affected organizations to develop processes before the Act came into effect in June 2019. This qualitative interview study investigates the perspectives of a multidisciplinary sample of twelve clinicians from a single metropolitan hospital during this implementation period. Maximum variation sampling was utilized to ensure breadth across discipline (medical, nursing, allied health), speciality, and stated level of support for the VAD legislation. Four key themes were identified from the interview data: preparing for the unknown, ethical diversity within the organization, building a respectful culture, and concerns about the inability of the legislated approach to capture clinical nuances. Overall, these clinicians’ workplace experiences during the implementation period were shaped by the ethical diversity within their organization and a sense of uncertainty about how the VAD legislation would integrate with the practical realities of their clinical setting. The concept of “ethical diversity” could be a useful one for supporting staff in an organization during a VAD implementation period.

## Background

Australian states have been part of an increasing international momentum around legalizing voluntary assisted dying (VAD). Various countries globally allow some form of voluntary assisted dying (Queensland University of Technology 2021), including Canada, several states in the United States, Belgium, Switzerland, and Colombia, with a variety of systems and eligible patient groups. In Australia, the state of Victoria passed legislation in November 2017 to legalize voluntary assisted dying, with a system primarily focused on patient self-administration of VAD medication and limited to terminally ill patients in their last months of life (State Government of Victoria 2017).^1^ At the time of writing, four other Australian states have since passed similar legislation.^2^ Voluntary assisted dying became legal in New Zealand from November 7, 2021, following a referendum in 2020 (New Zealand Government Ministry of Health—Manatū Hauora 2021). From an ethical perspective, this global momentum has led some ethicists to reflect that “[t]he debate in medical ethics about assisted dying has by and large moved on from the question of whether assisted dying is in principle morally defensible to the specifics of when it is morally defensible” (Downie and Schuklenk 2021, 662).

To be eligible for VAD in Victoria, a patient must have a prognosis of no more than six months or no more than twelve months in the case of neurodegenerative disease. Decision-making capacity and residing in Victoria are also eligibility requirements. The key features of the system are presented in box 1 (McDougall et al. 2020). During the parliamentary debate about VAD in Victoria, the proposed system was consistently presented as the “safest and most conservative” in the world (McDougall and Pratt 2020, 3). Patients are expected to self-administer the medication unless they are physically unable to do so. Two senior doctors are involved: a coordinating practitioner who manages the process, including assessing the patient’s eligibility and prescribing the VAD medication, and a consulting practitioner who provides a second assessment of the patient’s eligibility. In the period June 19, 2019, to December 31, 2020, 240 Victorians ended their lives using the VAD system (Voluntary Assisted Dying Review Board 2021).

**Box 1:**
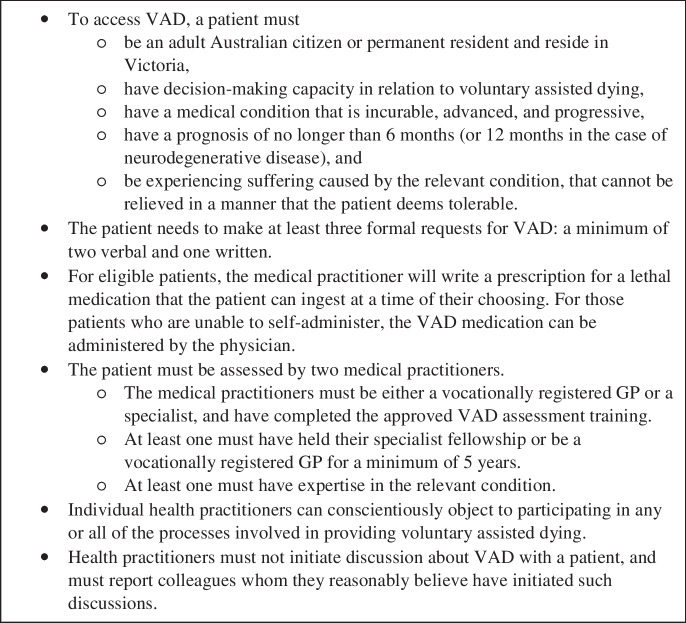
Key features of the Voluntary Assisted Dying Act 2017 (Victoria)

The substantial time period between the passing of the Victorian law and the start date for patient access to VAD was referred to as the “implementation period.” The parliamentary inquiry that precipitated legislative reform in Victoria had recommended that “[a]ny assisted dying legislation should include an eighteen-month period between Royal Assent and operation, to allow appropriate time to prepare for implementation on a practical and clinical level” (Parliament of Victoria Legal and Social Issues Committee 2016, xxxiv). The Victorian VAD legislation passed through Parliament on November 29, 2017, with a start date for patients to access VAD specified in the Act as June 19, 2019 (*Voluntary Assisted Dying Act 2017*; State Government of Victoria 2017). On April 28, 2018, the terms of reference for the government’s “VAD Implementation Taskforce” were published; these included “[e]stablishing guidance, support resources, and organisational service models” (State Government of Victoria 2018, 2).

This implementation period characteristic of the Victorian system has been replicated in other Australian states that have subsequently legalized VAD (Government of Western Australia Department of Health 2021; Tasmanian Government Department of Health 2021). Implementation periods have also been part of the legislative change process for VAD in, for example, Spain (Velasco et al. 2021; The Canberra Times 2021), Austria (Queensland University of Technology 2021; DIGNITAS 2020; Braun 2021), and New Zealand (New Zealand Government Ministry of Health—Manatū Hauora 2021; Roehr 2021). However, it is not a universal feature internationally, as the possibility of an implementation period is dependent on the process by which VAD becomes legally available in a particular jurisdiction (Roehr 2021). In Canada, for example, the prohibition on assisted dying was found by the Supreme Court of Canada to infringe the Canadian *Charter of Rights and Freedoms* (Downie and Schuklenk 2021); thus medical assistance in dying became available more swiftly and without the substantial planning period that the legislative process experienced in Victoria allowed (White, Willmott, and Close 2019). It is also worth noting that, within the VAD literature, the term “implementation” is not universally used in the sense of a planning period *prior to the start date for patient access*. However, that specific time window between legislative change and patient access is the focus of this study, and hence the term “implementation period” will be used in that sense in this paper.

In Victoria, the implementation period was a time of intense activity both within the relevant government department (the Department of Health and Human Services [DHHS]) and within the range of health and aged care organizations who would be impacted by the availability of VAD. In January 2019 with six months remaining in the implementation period, DHHS published the model of care pathways for healthcare organizations. Hospitals were able to choose from three different pathways, involving different levels of service provision in relation to VAD (Table 1) (State Government of Victoria 2019). At the time of data collection for this study in May-June 2019, the hospital involved had chosen to be a pathway A service, that is, to provide VAD.

**Table 1:** Model of care pathways (State Government of Victoria 2019)

Pathway A	“single service”	an eligible patient who requested VAD could be fully supported within the organization
Pathway B	“partnership service”	the organization “may support and facilitate the request and assessment process but will need to establish partnerships with other health services and refer people to other services to access appropriate specialists”
Pathway C	“information and support service”	will “provide information and/or referrals for people who want to request voluntary assisted dying and, where appropriate, continue to provide support to these people”

While there is a substantial body of empirical research on healthcare professionals’ views on VAD, and their experiences of providing or objecting (White, Willmott, and Close 2019; Rutherford 2020; Haining, Keogh, and Gillam 2021; Yoong et al. 2018; Blaschke et al. 2019; Karapetis et al. 2018; Fujioka et al. 2018; Beuthin, Bruce, and Scaia 2018; Bruce and Beuthin 2020), the existing literature on healthcare professionals’ views and experiences *during an implementation period* is very limited. Healthcare professionals’ experiences and views in an implementation period are potentially different from those prior to legislative change or those occurring once VAD is available in a health system. During an implementation period, a specific VAD regime is set and so clinicians are expressing views on a particular concrete VAD system rather than more abstract views on VAD in general. Further, during an implementation period, clinicians are experiencing an impending real change in relation to the health system in which they practise, rather than either a hypothetical scenario of legislative change or lived experience of VAD being legally available. To date, published data about this time period have primarily been limited to survey responses (McDougall et al. 2020; Fuscaldo et al. 2021; Booth, Eleftheriou, and Moody 2021; Sellars et al. 2021a; Snir et al. 2022), which provide important insights but limited detail and nuance in relation to participants’ views. The existing data indicate that support for the implementation of VAD may be lower among medical specialists, particularly palliative care specialists (Sellars et al. 2021a; Philip et al. 2021), than among other hospital staff (Fuscaldo et al. 2021; Sellars et al. 2021a). Implementation challenges are anticipated to range from the level of the individual clinician to that of the hospital or health system as a whole (McDougall et al. 2020; Booth, Eleftheriou, and Moody 2021). While the literature includes reflective pieces (Philip et al. 2021; Lee 1997; Le and Philip 2018) and non-empirical analyses (White, Willmott, and Close 2019; Johnston and Cameron 2018; Moore, Hempton, and Kendal 2020) focused on implementation periods alongside survey reports, a study of pharmacists (Woods et al. 2021) and a study of conscientious objectors (Haining and Keogh 2021) are, to our knowledge, the only interview studies examining practitioners’ views in an implementation period to date.

The field of implementation science has grown to address the complexity and difficulty of implementation, building on the insight that interventions fail to translate to patient care outcomes if not approached appropriately. Ideally, health services aiming to implement VAD should use existing implementation frameworks and should consider the following five domains (or similar): the intervention, the inner setting, the outer setting, the individuals involved, and the process by which implementation is accomplished (Damschroder et al. 2009). The current study focuses on the fourth domain, the individuals involved, because individuals have agency and the ability to make choices and, hence, wield power over and influence others in ways that could predictably support or hinder implementation of VAD. Understanding clinicians’ experiences in the implementation period is thus an important piece of the puzzle in VAD research.

As well as focusing on the implementation period specifically, a key aspect of the conceptual framework of this study was conceptualizing the individual clinician as part of a team, department, and organization in relation to VAD (Oliphant and Frolic 2021). Existing research on clinicians’ views and experiences in relation to VAD tends to focus on individual decision-making and practice within specific professional groups such as oncology (Yoong et al. 2018; Karapetis et al. 2018), palliative care (Blaschke et al. 2019; Wright et al. 2021), geriatrics (Munday and Poon 2020), nursing (Snir et al. 2022; Wilson, Oliver, and Malpas 2019), or pharmacy (Woods et al. 2021). Our focus went beyond participants’ individual decision-making to investigate their experiences as part of a team and an organization navigating this significant change in the healthcare landscape. The following research question guided the study: What are clinicians’ experiences and ethical views in the implementation period, prior to VAD becoming available in their hospital workplace? Our aim was not to evaluate the implementation process at this particular health service. Rather, we aimed to investigate the experiences of the multidisciplinary clinical staff in a range of teams and departments within an organization implementing VAD, including their own ethical position on VAD and their views on the Victorian VAD system as an impending reality in their workplace. Understanding health professionals’ experiences in this context has the potential to improve support for clinicians when VAD or other morally controversial changes in health policy are legislated.

## Methods

This interview study followed on from a larger mixed methods online survey of clinical staff across seven health services conducted from November 2018 to February 2019, during Victoria’s implementation period for VAD. The larger survey study aimed to collect data about the willingness of clinical staff to participate in VAD, in order to inform decision-making by the health services about which of the model of care pathways was the appropriate choice for their organization. The results of the larger survey study are reported elsewhere (Sellars et al., 2021a; Snir et al. 2022; McDougall et al. 2020).

Survey responses from the larger study were used in the sampling strategy for this interview study. The final question of the survey invited participants to volunteer for a qualitative interview, including noting their contact details. Using the pool of volunteers from this organization, potential interview participants were identified as those who had indicated in the survey that they look after patients with advanced incurable illness with a prognosis of less than six months, “daily” or “at least once per week.” Within this set, maximum variation sampling (Kitto, Chesters, and Grbich 2008) was used to recruit a group of interview participants that would include a range of disciplines, different views on the VAD legislation (support, unsure, oppose), and a variety of specialty areas based on information provided in the survey. Potential participants were contacted by email, with one reminder. A total of thirty-six volunteers were contacted and twelve interviews conducted.

The twelve individual semi-structured interviews were conducted in the period May 3 to June 12, 2019, with the implementation period ending on June 19, 2019. Sufficient rich data were collected to answer the research question, but saturation was not reached given the fixed end date for data collection at the end of the implementation period. Participants covered medical specialists, junior doctors (fellow, registrar), nursing, social work, physiotherapy, and spiritual care staff. Participants worked in oncology, palliative care, geriatrics, psychiatry, surgery, respiratory, emergency, and general medicine (Table 2). Interviews lasted between thirty-one and fifty-five minutes, with a mean duration of forty-one minutes. Each interview explored the participant’s:

- own ethical position on VAD’s legalization in Victoria, including the reasons for their view
- anticipated practice in relation to VAD when legal in Victoria and the likely impact on their patients of Victoria’s legislative change
- experiences in their department during the implementation period, including discussions with colleagues and potential for conflicting views
- views on organizational culture around VAD including non-participation, and the relationship of VAD to palliative care within the hospital.

**Table 2:** Participants, *n*=12. Profession and department have been reported separately to protect participant confidentiality

Distribution according to profession	Senior medical	5
	Junior medical	2
	Nursing	2
	Physiotherapy	1
	Social work	1
	Spiritual care	1
Distribution according to department	Palliative care	2
	Medical oncology	2
	Geriatrics	1
	Psychiatry	1
	Surgery	1
	Respiratory	1
	Emergency	1
	General medicine	1
	Spiritual care	1

All interviews were conducted by RM. RM was not employed by the hospital at the time of conducting the interviews but in her role as a university-based ethicist had contributed on the organization’s VAD working group and had presented survey results to the wider clinical community at this organization. One of the interview participants was a member of the organization’s VAD working group. MS worked as a researcher within the organization at the time of data collection; he was not involved in recruitment and transcripts were de-identified prior to his analysis of the data. BP was a university-based ethicist, who had not had contact with the clinical community at this hospital. The team were neither advocates nor opponents of VAD; the focus of our research interest was the ethical implementation of this legislated change in the Victorian health system.

All interviews were audio-recorded with the participant’s written consent and professionally transcribed. The accuracy of the transcripts was verified by RM . Data were analysed thematically (Braun and Clarke 2006). The first round of analysis involved analysing the data for a broad thematic description of the data set. MS coded all twelve transcripts and developed a draft coding framework. Independently, RM and BP each coded six transcripts, then the draft coding framework was refined by the group. A second round of analysis was then undertaken, developing a more nuanced and detailed account focusing on implementation experiences and ethical views. RM and MS reread and recoded the data, and a final set of themes and categories was agreed in ongoing discussion with BP. Investigator triangulation was used throughout as themes were revised at each stage of the process.

## Results

**Fig. 1:**
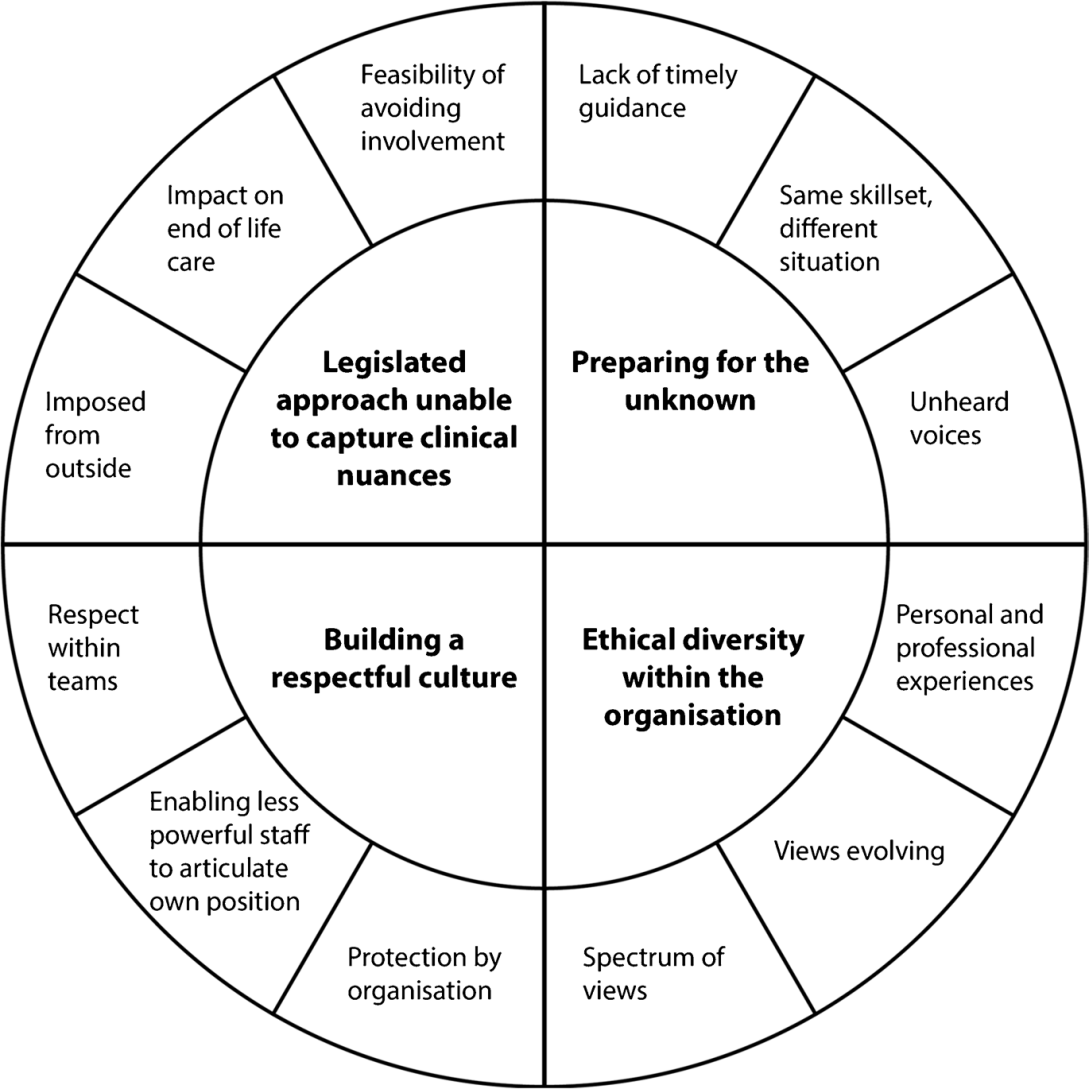
Themes and categories

Four themes were identified from the interview data: preparing for the unknown, ethical diversity within the organization, building a respectful culture, and an inability of the legislated approach to capture clinical nuances. The themes and categories are presented in Fig. 1. Overall, these clinicians’ experiences in the implementation period were characterized by ethical diversity and practical uncertainty about the way in which the new Victorian VAD system would work in their clinical setting. Ethical diversity was perceived within the organization and in individuals’ own evolving views on VAD. To protect participant confidentiality in the descriptions of the four themes below, identifiers have not been used to link quotes to participants. All participants are quoted within the text. Where multiple quotes are used to evidence a category, the quotes are from different participants.

### Preparing for the Unknown

Participants were preparing for the reality of VAD in various ways but with a sense that it was not possible to know fully how VAD would impact their workplace until after the start date. Their experience during the implementation period was therefore one of preparing for the unknown.

Some clinicians were waiting for information about processes. Their ability to prepare was limited by a lack of timely guidance during the implementation period. As one participant said, “at the moment I think we’re probably all a bit in the dark.” Some participants expressed concern that information from the organization and from the relevant government department was only becoming available late in the implementation period: “it’s only very recently that the actual details have become apparent [from the department] … I think it’s all a bit late in the piece.” There was a sense of clinicians waiting for direction:

Everybody on the ground has been very much kind of waiting to be given direction … Even in my own department we as a group of doctors probably haven’t sat down together yet, we’re kind of waiting to be directed, well here’s all the processes, now sit down and talk about them … We just haven’t taken it upon ourselves, it seems like a too big a thing, so yeah, I think that’s what it is, it’s waiting for guidance from above.

While many participants were waiting for guidance, in contrast, some participants felt well prepared, describing education sessions and/or discussions within their teams about how they will approach patients who raise VAD.

I think that the education that we’ve had so far has been really, really good … [Educator] has been able to sort of provide the [wording], okay so if you get asked this, this is a few options of how you could respond.

This type of practical education was highlighted as necessary by clinicians who had not yet had sessions in their departments. Overall, those who were engaged in training felt it was very practical and focused on first conversations with patients raising VAD across a wide range of clinical roles. However, some participants also indicated that there were areas of the hospital with unmet need for this type of education and support at the time of data collection, particularly for staff in areas that may encounter VAD requests but were not anticipated to be highly impacted by the availability of VAD.

Participants’ preparation for the unknown was aided by linking VAD to their existing skillset. Some participants described VAD as a new situation in which their existing clinical skills could be applied. While VAD was seen as a very significant change––“it’s a big cultural shift in terms of the way we’ve been brought up as doctors”––it was also seen as utilizing some of their existing skills. Specific skills such as responding to desire-to-die statements or conducting capacity assessments were highlighted, described in terms such as “that’s my job, normal stuff.” This experience was shared across medical and non-medical roles. As one allied health professional noted: “I think because of the work that I do, that I can jump in and do the work that’s required [for VAD].” For some participants, it was approaching VAD with the same attitude or stance of patient-centredness as they did in their usual work to date: “that’s how we work normally anyway, we know what our own stuff is, we don’t bring it in.”

There appeared to be a consensus that clinicians’ existing skill sets encompass many aspects of care relating to VAD. However, the lack of certainty about clinicians’ own roles in implementing VAD challenged confidence in this regard for some participants. There was a sense that skills could be applied but sometimes in the context of uncertainty about exactly how VAD-related care would be delivered in their setting.

Some participants expressed the view that the focus had been on engaging senior medical staff during the implementation work at their organization, with the unintended effect of excluding other relevant staff. There was concern expressed about unheard voices within the organization, particularly voices from non-medical disciplines and more junior levels of the medical hierarchy.

I think the focus is largely on, you know, the doctor involved in the process but that really, in the scheme of things, is such a short part of the entire voluntary assisted dying process and it’s all of the other support staff that are going to be impacted.

I’ve been a bit disappointed that we haven’t actually been engaged in this … there’s been a lot of high level stuff happening, but it feels, I mean it’s June, it’s [starting] next month … it feels like it hasn’t been given enough oxygen to actually—for people to really discuss it and think about what it means in terms of practice.

There was concern that not engaging more broadly could be problematic for the organization’s later provision of VAD care: “you’re not looking at the silent majority as it often is––they can either be struggling or actually scupper a process, you know people that are actually objecting but not saying it.”

### Ethical Diversity Within the Organization

There was a wide range of positions on VAD within the participant group. This was expected, given the sampling strategy. However, the interview data demonstrated a nuanced landscape of ethical views on VAD within this participant group, beyond the three categories of “support,” “unsure,” and “opposed,” which were the available options in response to the survey question. Within this group of clinicians, positions ranged from being willing to prescribe the VAD medication to being unwilling to refer a patient who requested VAD. Additional dimensions such as a pre-existing relationship with the patient and the patient’s ability to self-administer the VAD medication were also central to some doctors’ willingness to be involved. The various individual ethical positions are illustrated by the quotes in Table 3, covering both medical roles in VAD (e.g., coordinating practitioner) and other roles in VAD related care (e.g., providing information in response to a request, psychosocial support for VAD patients and their families). Some participants were strongly motivated by a specific consideration, such as witnessing patient suffering. Others cited multiple considerations—goals of medicine, patient-centred care, patient autonomy/rights, dignity, community impact—which were balanced in different ways by different participants.

**Table 3:** Range of participants’ ethical positions on involvement in VAD-related care

Not willing to participate or refer	“I am opposed to voluntary assisted dying in all circumstances … I see referring on as I guess part of that causal chain towards voluntary assisted dying, and I wouldn’t be comfortable doing that. I would be comfortable telling the patient to seek advice from their treating team, which is what I expect that I will do”
Not willing to participate but willing to refer	“I would not be myself involved in the actual process but I’m more than happy to give information or do that initial screen [for eligibility] … I think it should just be like abortion. Even though I don’t agree with VAD, it should just be like abortion where there is a legal obligation to refer because if it’s legal, it’s legal, and you know whether or not you agree with it isn’t the point” “I have reservations about voluntary assisted dying … and in my practice I feel like I would find it quite difficult to, to do … to make that cultural shift or the paradigm shift of actually initiating the end of someone’s life … I think I would be comfortable enough to have that first sort of exploratory discussion and say are you really, you know is that really what you want, but I think that’s probably where I’d then stop”
Prefer not to participate but willing to refer	“I’d be prepared to make the referral to someone who was able to do it, but I would personally feel quite uncomfortable about, about doing it [being one of the practitioners] … You know philosophically I can rationalize the importance or the value of the role of it under certain circumstances but I can’t see myself, I can’t see myself doing it. You know if I had to, I probably would but if, you know … if the circumstances were such that there was no other option and the patient was requesting it and in my view you know well all the criteria were met and there was no, you know, that the setup wasn’t such that there were others prepared and able to do it, yeah then—but given the choice of being able to refer someone, I’d much rather be the person who supports the patient and says if this is what you want then I’m happy to introduce you to someone who can help you rather than to be personally responsible for it.”
Currently unwilling to participate but may later be willing to be a coordinating practitioner for patients that they have cared for long term	“I don’t intend when I get my letters to like you know do the training and sign up. I think it would very much probably depend on if I met a patient that I felt, that I guess I heard their story, looked after them, and I guess to feel comfortable that everything else, and but that was still this, something that they really wanted and everything else had been explored properly, then I might consider it.”
Willing to be a coordinating medical practitioner only when patient plans to self-administer the medication	“I’m happy enough to be one of the practitioners involved, I know I’m not going to be somebody who will want to administer the medication.”
Willing to be a coordinating practitioner for long-term patients but pragmatic barriers	“From my point of view, I’m very supportive but quite frankly this sort of six-hour time period or something that you have to go through to get sort of certified is a barrier for me … Yes in principle [I’m willing to be co-ordinating practitioner] … But at the moment I really don’t see in terms of pragmatically and in terms of having to make time during the day or the time to get the certifications … [I’d] probably be much more happy sort of filling the papers and being one of the clinicians involved if it’s a patient I’ve been particularly involved with over the years and this is about their wish to, to, for me to help them in that. Someone who I don’t, haven’t really met before …, particularly a patient from another disease area I feel a bit reluctant about that, it’s a bit anonymous, sort of depersonalized.”
Willing to provide capacity assessment	“I’d happily discuss it [VAD] with them, but I’d direct them to speak to their treating clinician for whatever the condition was. So, I kind of see my responsibility as having at least some knowledge of the framework and what they need to do to kind of progress things if I’m asked, and obviously if asked you know make a comment on capacity if that was an issue.”
Willing to support VAD patients and families— non-medical role	“So, for me it’s like actually it’s not up to me to make the decision around it, there’s bigger things at play here, so in terms of whether I support it, I just go well if that’s what our government has decided and that’s what our hospital is doing then I actually will support that and work with it however we’re asked to” “we’re probably always going to direct it back up through the medical team … it’s just another option that patients have, and I think that that’s a good thing … I think it’s just like anything that, in our workplace is that we do have to respect what the patient’s decision is you know, and we talk about patient-centred care, and this is a decision they’ve come to, and there’s clearly defined steps or hoops that the patient has to go through to be able to actually get to that stage. So, and I think if they have gotten to that stage, then we need to respect to what the patient wants” “I’m in favour of it [VAD] … I just feel it’s a person’s right to be able to make a decision about the end of life and how they want to finish their life, within some very strict guidelines, which appear to be very strict within the Victorian legislation.” “I am in full support of voluntary assisted dying.”

*“Allied health” covers clinical roles outside medicine and nursing, such as social work, physiotherapy and spiritual care

Many participants had thought deeply about the ethics of voluntary assisted dying and had developed their current view over many years. Some participants perceived their ethical position on VAD as a permanent commitment, while others saw it as fluid and potentially changing:

Whenever I’ve heard about it [VAD], I’ve always thought oh yeah, I’m in favour of that.

I don’t think any of my views have ever been fixed. They’ve always been a bit fluid, so I’m sure that as it [VAD] comes in, you know, my positions will evolve.

Professional experiences in clinical work provided important reasons motivating participants’ positions on VAD, including witnessing patient suffering.

It’s been definitely my clinical experiences that have let me think, you know, this should be an option for patients.

While witnessing suffering was a professional experience that motivated support for VAD, for other participants there were aspects of their professional experiences that motivated concerns about VAD. These included witnessing potential coercion and poor-quality palliative care.

I guess we see how vulnerable our population of patients are, and I definitely have seen coercion from family members about all sorts of things.

What my clinical experience has shown me is that when it [palliative care] is done well it can be really good … the requests for voluntary assisted dying coming from a place of poor symptom control and poor management.

Personal experiences as family members had also shaped participants’ ethical positions.

One of my big reasons as just a general member of the community is I’m heavily involved in the disability community outside of work ... I’ve got two [family members] with [name of disability] who are very active in the community actually, probably more active and contribute more than I do. If we’re allowing that certain people who have certain illnesses or conditions, we’re allowing that they are better off dead then that––and it’s hard for me to explain but it does impact our disability community, just that cultural shift towards saying that at some point you know there is people in the community that we’re accepting that they’re better off not living.

Over the last two years I’ve had both my parents diagnosed with terminal illness and my mother died last year, late last year, yeah, so I’ve been through it … we’ve actually had some discussions about VAD within the family … and if he [father] chooses to go down that way, I’d be very supportive of it.

All participants had sophisticated ethical positions on VAD that had evolved over time, based on their personal and professional experiences.

Just as there was a wide range of positions on VAD within this group, participants also reported that there was similarly a wide range of positions on VAD amongst the clinical staff in the organization.

So I said [to our team] well how do you guys all feel because we need to know, and you know it’s not going to go further than this room … They’re quite open, some of them say well I’m not really sure; others say, you know, I’ve got some who’ve got a background in vet science who just went well, of course. And others who are saying I’m fine as long as I don’t have to actually do anything in terms of be part of the decision-making or in terms of having to counsel people.

In general, I think yeah some people are comfortable with it, some are uncomfortable with it. There are certainly some who are going to be very reluctant to be involved directly I think.

Again, the variety of positions on VAD were seen as more nuanced than simply participators and objectors: “It’s degrees, you know, happy to be involved in assessment and usual care and giving information but not to actually do it.” Ethical views on VAD more generally as well as views on specific aspects of the Victorian system combined to create this nuanced variety of positions.

Three participants specifically used the language of “diversity” to describe the range of positions held by clinical colleagues in relation to VAD. In each of these interviews, this was the first occurrence of the word “diversity,”—that is, participants were not reflecting language used previously by the interviewer.

Well, I think one thing that the organization’s been quite good at, probably since [name of CEO] has been around, is a bit more embracing diversity. It’s very much you know on the agenda, and it is within [department], we have a diversity group, so I sort of see this as kind of coming under that. Although it is law, it’s diversity, people have different views, so their, everyone’s, views should be respected.

There’s clearly a diversity of opinion among the junior staff as well.

One participant specifically framed moral diversity as a strength in the health professions:

I think it’s really important to have that you know I guess moral diversity. I think that’s what keeps medicine rich really is, is having people with different views that are relevant in various roles rather than us all being you know passive, I don’t know what’s the word, demoralized agents just enacting a patient’s will.

Organizational acknowledgement of ethical diversity may be an important aspect of implementation— being explicit about types of ethical positions that are acceptable. Another participant, who was not willing to be a coordinating or consulting practitioner for VAD, similarly highlighted the importance of being engaged in VAD discussions in the organization:

It’s actually important for people like me to be involved because you know we can do all the usual stuff that by and large makes the request disappear, so if we object then logically you’d think actually you want to be involved more. But I would never do it purely to dissuade them [patients] from that, but I would see them no longer wanting it as a success because you’ve managed to address the suffering. But not ideologically because I don’t want them to do it, just because that means they don’t need it anymore.

These insights suggest that ethical diversity within the clinical staff is a potential strength within an organization where VAD is available. The organization’s ability to acknowledge and accept ethical diversity was suggested as a means of potentially improving staff experience at work and the quality of patient care offered.

### Building a Respectful Culture

Related to ethical diversity, a further theme in the interview data was the need to build an organizational culture that was respectful of staff members’ different views on VAD. While ethical diversity was seen as a potential strength, it was also seen as creating challenges during the implementation of VAD that required strong respectful leadership within the organization.

So, I think the organization needs to be very clear that they will support staff who conscientiously object and that I guess people’s values are going to be different. I’m not prepared to be involved in any of that process, others might be different and so I would want to know that [hospital name] will support me wherever I draw that line.

The culture is one of respecting people’s views, so whether it’s positive or negative, it’s okay. And I think that’s the way they’ve [the organization] approached it, because they haven’t just gone this is law, we’ve got to do it. They said this is law, but you know we realize that some people will be against it and won’t be for it—that’s okay, we’re going to work around that and not be derogatory towards people who are against it.

Some participants expressed this in terms of the organization “protecting” staff, encompassing both those who choose to be involved in VAD-related care and those who object. Various features were highlighted as important to protecting staff, such as rostering, maintaining privacy, and peer support:

The ward, the hospital, is very conscious about protecting their staff and looking after their staff. I think that we’ve got a really good culture of that, so I think that if this is something that’s really distressing somebody, that it’s actually okay to roster them away, and so we will protect them.

I’m not convinced that there’s been sufficient thought put on how the clinicians involved in this will be supported from a number of fronts … how you protect the privacy of the individuals involved [in providing VAD]. And how they get peer support … for a greater range of doctors to be taking on that sort of responsibility without any structure around support I think is potentially problematic.

While there was consensus that a respectful and protective organizational culture was needed, some participants perceived the hospital as achieving this in relation to VAD and others did not.

Some participants spoke of the need for less powerful members of staff to be specifically supported by the organization to engage in ethical reflection and dialogue about VAD. Cognizant of the hierarchical nature of clinical work and the power dynamics amongst different types of staff, for these participants, a respectful organizational culture included enabling junior doctors, nurses, and other staff to discuss, develop, and articulate their own views on VAD.

Hospital hierarchy, particularly medical hierarchy is so ancient and enshrined and junior doctors are under pressure all the time, in various often quite subtle ways, to conform to their seniors’ sort of opinions and what they want … A lot of the hierarchy is also based on wanting respect, respect from your seniors, potentially, and modelling from them as clinicians. There’s a huge pressure to get into training programmes and … be respected by your bosses … So, when there’s this really contentious kind of issue that you really need to look inside yourself and it is kind of like an ethical thing, I think that’s quite hard. Because people are really clouded by this kind of often loyalty and respect, and they want to model themselves on a senior. And there’s a real tension between that, yeah, I think there will be a real tension between that and necessarily really looking inside yourself and saying what is my position on this … your own principles kind of thing.

For these participants, part of building a respectful organizational culture was providing opportunities and guidance for less powerful members of staff to reflect on the ethics of VAD and articulate their own individual ethical position. They posited the need for educational opportunities that included this type of ethical dialogue, rather than simply factual or legal or clinical information.

People have a bit of an instinct about it but then without the knowledge or the education perhaps unable to verbalize why they feel the way they feel … they don’t really know why they feel the way that they do, and to then be able to explore that with them, that is what is far greater than you know a ten-minute PowerPoint on the legislation.

A respectful culture was one in which staff were encouraged to *develop* their own ethical views on VAD, not simply one in which a range of expressed views were tolerated.

At the team level, participants raised the importance of prioritizing respect amongst colleagues in the context of diverse views on VAD. The sense of facing the change as part of a team was clear:

Initially, I was like, when I didn’t know much about what it, how it was going to work it was like okay what does this––and particularly for my staff I was thinking, what will this mean for us?

**I’m a bit nervous about, so the first one is, well, the team. How am I going to communicate this now to the team that this is the direction that we’re going in?

The importance of respect within the team was emphasized.

There are people in my team that have different religious views to me and political views to me and I can still have a conversation with them about it or around it, and I can respect their values and views, and I’d hope that people would have the, we’d all have the same courtesy around this, but it’s new and we don’t know.”

Others focused on respectful *behaviour* specifically, rather than respect in a cognitive or attitudinal sense.

You don’t have to respect the other person’s opinions, but you have to behave respectfully to the other person. She [presenter running a departmental session on VAD] changed it, she goes six months ago I told you guys you have to respect everyone and then now I’m going to change it to say you don’t have to respect their opinions, but you have to behave respectfully towards them, which everybody laughed and then thought you know that’s a very nice way of putting it.

You can have that conversation without being it a conflict, right. So, I think if it’s handled in a respectful way, it shouldn’t be a problem.

The need to face this legislative change as a team guided by respect was central to participants’ experiences.

### Legislated Approach Unable to Capture Clinical Nuances

In various ways, participants expressed the concern that the legislated approach to VAD was unable to capture the nuances of the clinical environment in which VAD-related care would actually be provided. Participants perceived the VAD system as imposed on clinicians from outside their professions.

One of the sort of sentiments I’ve heard … from clinicians in general is that it’s something that’s been kind of like foisted on us through legislation without much consultation.

Additionally, VAD was also seen as being imposed on their *organization*, even amongst clinicians who were supportive of VAD.

I think it’s a big change in that it’s been legislated from outside, it’s not like something like a strategic direction that [name of hospital] are going in. Let’s do this because it’s part of our, you know, whatever, our mission statement, and it’s not that, it’s something that’s come from outside that we have to adapt [to].”

Being imposed from the outside, the legislated VAD system was seen as having features that fit poorly with clinical reality and current health system organization. Existing confusion within clinical settings about the role and nature of palliative care could potentially be further compounded by VAD.

I guess from a health professional’s point of view the impact that that shift has, and the confusion that that creates I guess within medicine, I don’t think we can downplay the seriousness of the issue … There is already a lot of misunderstanding around end-of-life care and we are adding to that in a way, rather than making it any clearer.

Demystifying normal dying and explaining that actually good end-of-life care does not kill someone is a large part of our job, and if suddenly being able to actively end someone’s life is a legal choice, whether or not it’s something that we’re actually doing or not, if patients and families know that it’s a legal choice then that line becomes a lot more blurry … People are already terrified of palliative care and if they know that people are actually having their lives ended then they’ll avoid it even more I think.

As well as impacting on patients’ understanding and trust of palliative care, there was also concern that the introduction of VAD could impact staff decision-making in relation to end-of-life care. Given the clinical reality of time-poor staff and under-resourcing, the availability of VAD was perceived as a potential threat to clinicians engaging in challenging and resource-intensive discussions with patients about distress and dying.

It’s just in the back of my mind that potentially VAD, it might reduce the impetus to actually explore that [patients’ distress] and drill down to that stuff, which is already very difficult and time consuming and resource intense.

What worries me about VAD is that it becomes a solution to a problem which should better be addressed in other ways but perhaps is much more difficult to address in other ways.

If the immediate response to any patient raising a VAD is immediately down the VAD pathway of taking this as a, you know, a real request for want of a better term, then you actually miss out on all the normal care because you get distracted by the [VAD] pathway.

However, this worry was not shared by all participants: “It wouldn’t change our practice in offering care.”

A further way in which the Victorian VAD system was perceived as a poor fit for the clinical setting was in regard to staff who wanted to avoid involvement. The Victorian legislation includes very strong protection for clinicians with a conscientious objection to VAD. There is no requirement to provide information to a patient who requests VAD nor to refer to another practitioner. However, participants highlighted that actually avoiding all involvement in VAD-related care would be difficult given the structures of hospital work: “will [staff] be able to not participate and how does that happen?” They felt that despite the law’s strong position, it was impractical in practice. Barriers to avoiding involvement are described in Table 4.

**Table 4:** Barriers to avoiding involvement in VAD

Rostering	“the risk of junior medical staff just through, like their roster being working with consultants that are involved” “It’s going to be very hard to roster around preferences.”
Working in a management role	“I think also for nursing management roles, for example my role, while I am on shift my role is supervision of the entire department … So, while a bedside nurse, I guess, might be able to say, hey I don’t want to look after that patient, I can’t unfortunately leave the entire department while that occurs, you can’t sort of tag team and just step out for a bit. And that’s something that I’ve thought a lot about over the last few months as to whether how that will impact me if I can’t truly conscientiously object because in some way, shape, or form in my role I will be responsible for that patient and that the voluntary assisted dying process, it will be occurring under my supervision. If there’s complications I will be as the nurse in charge, the one called in, I will be the one dealing with the family”
Hierarchical decision-making and culture	“The reality is that it’s the resident that gets called in the night, it’s the resident that writes the death certificate, it’s the registrar that you know mostly has the conversations with the family and the patient, and so if there’s, at a patient at a consultant level, a determination that this will proceed, how do you do it in a way that protects junior medical staff if they choose not to be involved? Because the way our hierarchical management system works is that the guidance and decision-making comes from consultants but the implementation is largely done by junior medical staff. So, I don’t know the extent to which the hospital has grappled with the mechanics of that.” “Medicine is a very hierarchical world and there’s huge power imbalances in that hierarchy. And it’s very, very, very hard for a junior doctor to not do what their seniors tell them to, or assume that they will … Part of the problem is this is not stuff that you can necessarily train people. You can tell them you can’t discriminate against it or there’s junior doctors and nurses who are not wanting to be involved, but people are people, and it would be very hard for the junior doctors to feel that they’re disappointing their boss.”

Participants, particularly junior doctors and nurses, identified a significant risk that clinical staff in these roles would be drawn into providing care that they were not comfortable with. Some participants saw it as likely or inevitable:

Practically speaking. I don’t think there’s any real way around it, I think people will be drawn in regardless.

Other participants did not.

I think we will be able to keep our juniors separate from it in any real sense … I think we’ll be able to keep them safe from it or comfortable with it.

The availability of dedicated specific staff for VAD was seen as important to enabling choice by clinicians about their involvement.

I think having that third party is very, very important, like the VAD programme manager is incredibly important for junior staff. I think a very clear organizational message that it is their choice, and I think having sort of a readily available people that can step in or that can manage things if they don’t feel that they can.

Completely avoiding involvement in VAD was anticipated to be very difficult in practice despite the legislative protection, reflecting gaps between the VAD legislation and the clinical environment.

## Discussion

In existing work on the Victorian VAD system, several authors have identified the key challenge of “translating aspects of the complex legislation into clinical practice” (Hempton 2021, 3575; see also White, Willmott, and Close 2019). This study provides more granular detail on the ethical aspects of this challenge from the perspective of clinicians in a health service where provision of VAD is imminent. The study highlights ethical diversity, practical uncertainty, and concerns about a poor fit between aspects of the VAD legislation and the clinical environment as defining features of the implementation period in this setting. Ethical diversity could create further challenges in the absence of a respectful culture.

Deepening conceptual understanding of the term “ethical diversity” is an important contribution of this study. In existing bioethical literature on a range of topics, the term ethical diversity tends to be used in the plain English sense of diversity, that is, a range or variety (Wright et al. 2021; Genuis and Lipp 2013; Hansen 2013). Previous studies on VAD have identified the varied spectrum of ethical positions within clinician communities. Rutherford et al. (2021, 200), for example, identify “the shortcomings of binary categories of support or opposition” based on their review of research on physicians’ attitudes to VAD. Moving beyond a characterization of clinicians as either participators or objectors is supported by these data, in which participants described a nuanced landscape of ethical views on VAD.

However, this study suggests an additional sense of “ethical diversity.” VAD views were linked to diversity in the sense of an organizational strength where there is inclusion and safety for staff of different genders, cultures, and sexualities (Garg and Sangwan 2021). While some previous writing has linked ethical diversity with religious or cultural diversity specifically (Irvine, Mcphee, and Kerridge 2002; Schmidtke and Cornel 2020; Harrison 2012), framing a spectrum of ethical views on VAD within a health service as a type of organizational diversity and inclusion is a new finding. This type of framing may be a useful tool for organizations navigating a VAD implementation period and offers an important area for future research. Our findings suggest that building a culture that is respectful of ethical diversity is an essential foundation prior to VAD legislation coming into effect. Shifting language from, for example, “conflicting views” to “ethical diversity” may offer some positive ways forward for organizations in developing a respectful culture. Ethical diversity offers a positive reframing of the range of morally acceptable views within an organization, highlighting the potential strengths that such a range might offer for patient care and staff experience.

A significant legislative change in a morally complex area such as VAD creates substantial uncertainties for clinicians, and such challenges can be exacerbated if clinicians feel a non-collaborative approach to implementation is adopted by decision-makers (Sellars et al. 2021b). In this study, many clinicians wanted more concrete guidance earlier in the implementation period and greater opportunity to work through the practicalities within their specific department or team. While the legislation set out the boundaries for the VAD system in Victoria, a great deal of practical detail and specific process work sat with the relevant government department. This created a substantial time lag in practical information becoming available to health services and subsequently to frontline staff and may also have contributed to study participants’ sense of VAD as imposed from outside the organization by non-clinicians. When these data were collected in the final weeks of the implementation period, many participants had significant concerns about how the system would translate to their clinical setting and impact their own work. The data also provide further support for previously identified specific practical issues such as the impact of VAD on end-of-life care (Fuscaldo et al. 2021; Philip et al. 2021; Waran and William 2020) and enabling conscientious objection (Haining, Keogh, and Gillam 2021; Booth, Eleftheriou, and Moody 2021; McDougall et al. 2021) or non-participation (Brown et al. 2021) in a hospital setting.

Particularly given the momentum around legalizing VAD in other Australian states and internationally, these participants’ experiences provide insights for organizations to consider in effectively engaging their staff during an implementation period. As well as timely concrete guidance, this study highlights the importance of opportunities for engagement across disciplines and departments. Given the resource constraints within health services, focussing VAD training on senior medical staff and/or a small number of departments likely to encounter VAD requests is understandable. However, this study suggests that a wide range of staff are seeking concrete guidance on responding when patients raise VAD. Further, it suggests that a broad conception of training is needed. Staff may value opportunities to engage in ethical dialogue about VAD, particularly junior staff and staff in non-medical roles. Enabling less powerful staff to develop and articulate their individual ethical position on VAD––that is, to do “moral identity work” (Wright et al. 2021, 7; Sellars et al. 2021b)––may be an important element of a respectful organizational approach to VAD in an implementation period. Reflective models (Oliphant and Frolic 2021; Pesut, Thorne, and Greig 2020) have the potential to support this ethical reflection and dialogue. Overall, significant resourcing for a broad, multifaceted education programme early in the implementation period is needed.

The study has several limitations. The data reflect the experiences of twelve clinicians within a single health service, and therefore cannot be generalized. The variation within the sampled clinicians is a strength for understanding staff experiences within this health service; however, implementation period experiences for staff in different health services (within Victoria and in other jurisdictions) may differ substantially. The findings capture implementation period experiences within the Victorian system specifically and may not apply straightforwardly to other geographical locations with different systems for VAD and for healthcare delivery more broadly.

## Conclusions

An implementation period presents an ethically important opportunity for health services navigating a morally controversial change in health policy. Understanding Victorian clinicians’ experiences during the VAD implementation period offers ways in which implementation periods may be used effectively and staff experience improved. Overall, these participants’ workplace experiences during their VAD implementation period were shaped by the ethical diversity within their organization and a sense of uncertainty about how the VAD legislation would integrate with the practical realities of their clinical setting. Concerns about a poor fit between the clinical setting and the legislation raise further questions of policymakers’ ethical obligations when drafting healthcare policies and laws. The concept of “ethical diversity” could be a useful one for engaging staff in an organization during a VAD implementation period (or in relation to other morally controversial changes in practice), given its strengths focus. With momentum around legalizing VAD in other Australian states and internationally, these participants’ experiences suggest the need for timely guidance, opportunities for engagement throughout the health service, and resourcing for a broad multifaceted education programme early in the implementation period.

## Footnotes

1 *Voluntary Assisted Dying Act 2017* (Vic).

2 *Voluntary Assisted Dying Act 2019* (WA); *End-of-Life Choices (Voluntary Assisted Dying) Act 2021* (Tas); *Voluntary Assisted Dying Act 2021* (Qld); *Voluntary Assisted Dying Act 2021* (SA).

## Declarations

The protocol of this study was approved by the Human Research Ethics Committee of the participating health service. The name and reference number have not been included, to protect confidentiality. Written informed consent was obtained from all participants in this study.
